# Chlorin e6-Conjugated Mesoporous Titania Nanorods as Potential Nanoplatform for Photo-Chemotherapy

**DOI:** 10.3390/nano14110933

**Published:** 2024-05-25

**Authors:** Estefanía Vélez-Peña, Verónica A. Jiménez, Joaquín Manzo-Merino, Joel B. Alderete, Cristian H. Campos

**Affiliations:** 1 Departamento de Fisicoquímica, Facultad de Ciencias Químicas, Universidad de Concepción, Edmundo Larenas 129, Casilla 160-C, Concepción 4070371, Chile; evelez@udec.cl; 2 Departamento de Ciencias Químicas, Facultad de Ciencias Exactas, Universidad Andres Bello, Sede Concepción, Autopista Concepción-Talcahuano 7100, Talcahuano 4300866, Chile; veronica.jimenez@unab.cl; 3 Facultad de Ciencias Químicas, Benemérita Universidad Autónoma de Puebla, Puebla 72570, Mexico; jmanzomerino@gmail.com; 4 Instituto de Química de Recursos Naturales (IQRN), Universidad de Talca, Avenida Lircay S/N, Casilla 747, Talca 3341717, Chile

**Keywords:** mesoporous titania nanorods, Chlorin e6, photodynamic therapy, combined therapy

## Abstract

Photodynamic therapy (PDT) has developed as an efficient strategy for cancer treatment. PDT involves the production of reactive oxygen species (ROS) by light irradiation after activating a photosensitizer (PS) in the presence of O_2_. PS-coupled nanomaterials offer additional advantages, as they can merge the effects of PDT with conventional enabling-combined photo-chemotherapeutics effects. In this work, mesoporous titania nanorods were surface-immobilized with Chlorin e6 (Ce6) conjugated through 3-(aminopropyl)-trimethoxysilane as a coupling agent. The mesoporous nanorods act as nano vehicles for doxorubicin delivery, and the Ce6 provides a visible light-responsive production of ROS to induce PDT. The nanomaterials were characterized by XRD, DRS, FTIR, TGA, N_2_ adsorption–desorption isotherms at 77 K, and TEM. The obtained materials were tested for their singlet oxygen and hydroxyl radical generation capacity using fluorescence assays. In vitro cell viability experiments with HeLa cells showed that the prepared materials are not cytotoxic in the dark, and that they exhibit photodynamic activity when irradiated with LED light (150 W m^−2^). Drug-loading experiments with doxorubicin (DOX) as a model chemotherapeutic drug showed that the nanostructures efficiently encapsulated DOX. The DOX-nanomaterial formulations show chemo-cytotoxic effects on Hela cells. Combined photo-chemotoxicity experiments show enhanced effects on HeLa cell viability, indicating that the conjugated nanorods are promising for use in combined therapy driven by LED light irradiation.

## 1. Introduction

Cancer remains a severe public health problem. According to GLOBOCAN, 20.0 million new cases worldwide and 9.7 million deaths derived from cancer in 2022 [1]. Though some treatments show satisfactory results, there is an urgent need for new technologies for therapy that produce better therapeutic responses against oncological diseases. Phototherapy has arisen as a favorable and less invasive alternative in cancer treatment, with high selectivity toward the diseased lesion (e.g., tumor) by limiting light irradiation to that area and fewer side effects [2]. It involves a phototherapeutic agent that can induce the death of tumoral cells under light irradiation without causing considerable damage in the dark. Photodynamic therapy (PDT) is a type of phototherapy based on the production of reactive oxygen species (ROS) after the irradiation of a molecular photosensitizer (PS) at an explicit wavelength in the presence of O_2_ [3]. ROS inhibits cell proliferation by nonspecific oxidative reactions [4]. Major drawbacks for current molecular PS used in PDT are the risk of skin photosensitivity and the non-specific accumulation of hydrophobic PS in the skin and eyes after the treatment [5]. This raises the need to develop new smart nanodevices that can overcome the limitations of PDT and enhance its effects with other therapeutic approaches in what is known as combination therapy. Nanomaterials are an excellent option for combined therapies, because they merge the well-known properties of nanoscale systems for PDT application. Among different photoactive nanomaterials, TiO_2_-based systems are good candidates for those purposes due to their chemical stability, biocompatibility, and optical properties [6]. From the PDT point of view, TiO_2_ is a semiconductor with photodynamic activity under UV irradiation, which has very limited penetration into human tissues and induces severe non-specific damages [7]. Several strategies have been developed to expand the light absorption capacity of TiO_2_ towards visible light. One of these approaches is the surface sensitization of TiO_2_ nanoparticles with organic PS (or dyes) through either covalent or non-covalent coupling [8]. Surface modification with porphyrins has raised interest, since it can significantly modify the absorption limit of TiO_2_ from the UV region to the visible spectrum. Chlorin e6 (Ce6) is a porphyrin-based photosensitizer with well-known phototoxic activity against various cancer cell lines under visible light irradiation [9]. Zhao et al. developed multifunctional chitosan-based micelles with a hydrophobic core in aqueous media, which serve to load Ce6 for magnetic resonance imaging-guided PDT. The results indicated effective MRI-guided PDT in 4T1 cells and an in situ 4T1 tumor model [10]. Other studies have immobilized Ce6 onto TiO_2_-based nanoplatforms for PDT. For example, Youssef et al. demonstrated the efficacy of Ce6 systems covalently coupled to TiO_2_ nanoparticles in PDT applications using U87 glioblastoma cells. Irradiation with a 652 nm diode laser system decreased cell viability by 89% in cells contacted with 200 μg mL^−1^ dispersions of the nanomaterials [11]. Jiao et al. reported the use of partially reduced TiO_2−x_ NPs and subsequent covalent conjugation of Ce6, which enable—by NIR laser radiation—combined photothermal therapy and a PDT effect in both cellular level and in animal models [12].

Current challenges in PDT have focused on combining light-mediated strategies with other therapies, such as chemotherapy, to improve the treatment efficacy through photo-chemotherapy (PCT) [13]. Typically, PDT in combination with chemotherapy using inorganic nanocarriers has been found to be beneficial. However, most reports on PDT applications using TiO_2_-based materials focus on using non-porous nanoparticles, which have a limited drug adsorption capacity. To overcome this drawback, multiple studies have advanced in using mesoporous TiO_2_-based materials as nanocarriers of therapeutics [14,15,16]. For example, our previous reports have evidenced the promising properties of mesoporous TiO_2_ nanotubes for drug delivery applications using different model chemotherapeutic compounds [17]. The recent report by Jian et al. has inspired us to propose the design of photosensitized mesoporous TiO_2_-based materials with suitable optical properties for PDT under visible light and high drug adsorption capacity to enable their use in combined PCT.

In this work, titania nanorods (TiO_2_NRs) were prepared using hydrogen titanate as precursor materials, which were calcinated at a controlled temperature and surface conjugated with Ce6 through assisted-silane covalent coupling. Ce6-conjugated NRs were characterized and evaluated for potential applications in dual PCT, taking advantage of the drug adsorption capacity of the mesoporous NRs and the photodynamic activity of Ce6. PDT activity was evaluated through phototoxicity experiments against HeLa cells under visible LED light irradiation. Chemo-cytotoxicity experiments were performed by incubating HeLa cells with solid suspensions of the Ce6-conjugated NRs preloaded with doxorubicin (DOX) as a chemotherapeutic model. Finally, the materials were evaluated in combined PCT experiments by exposing HeLa cells to suspensions of DOX-loaded materials, followed by visible LED light irradiation. Our findings revealed enhanced antiproliferative effects through the combined strategy, highlighting the potential of Ce6-conjugated mesoporous TiO_2_NRs as nanoplatforms for PCT applications.

## 2. Materials and Methods

### 2.1. Materials

TiO_2_ nanoparticles (nanopowder, particle size < 25 nm, anatase, surface area by BET 45–55 m^2^ g^−1^) and N,N′-dicyclohexylcarbodiimide (DCC) were obtained from SigmaAldrich^®^ (St. Louis, MI, USA). Sodium hydroxide (NaOH), nitric acid (HNO_3_, 67%), toluene, 3-(aminopropyl)-trimethoxysilane (APTMS), acetone, dimethyl sulfoxide (DMSO), and triethylamine (TEA) were provided by Merck^®^ (Rahway, NJ, USA). N-hydroxysuccinimide (NHS), Singlet Oxygen Sensor Green (SOSG), and hydroxyphenyl fluorescein (HPF) were sourced from Thermo Fisher Scientific (Waltham, MA, USA). Chlorine e6 (Ce6) was furnished by Cayman Chemical (Ann Arbor, MI, USA). HeLa cell lines were purchased from ATCC (Manassas, VA, USA) and maintained at 37 °C with 5% CO_2_. CellTiter 96^®^ Aqueous One Solution Cell Proliferation Assay (MTS) was supplied by Promega (Tokyo, Japan). All reagents were used as received without further purification. The LED light irradiation was provided for a lamp of 200 W with wavelength from 400 to 780 nm with two maximums of emission at 450 nm and 590 nm as displayed in the Supporting Information.

### 2.2. Synthesis of TiO_2_ Nanorods

Hydrogen titanate nanotubes were used as precursor materials, which were prepared using a hydrothermal method previously reported by our research group [17]. The obtained precursor nanotubes were calcined from 25 °C to 100 °C at 5° min^−1^, then 100 °C to 500 °C at 2° min^−1^, and an isothermal treatment to 500 °C for 30 min using 30 mL min^−1^ in static air atmosphere to obtain pristine TiO_2_ nanorods (TiO_2_NRs).

### 2.3. Preparation of TiO_2_NRs Coupled to Ce6

Pristine nanomaterials were surface functionalized with Ce6 using APTMS. An amount of 1.0 g of the TiO_2_NRs was dispersed in 50.0 mL of dry toluene, then 3.7 mmol of APTMS was added to the dispersion and refluxed for 24 h in a dry atmosphere. The nanostructures were centrifuged and exhaustively washed with 20 mL of toluene (×3) and 20 mL of acetone. Then, the TiO_2_NR with -NH_2_ surface groups (APTMS-TiO_2_NRs) were dried in a vacuum oven at 50 °C for 24 h. In parallel, Ce6 activation was carried out according with previous reported protocols [18]. A total of 0.05 mmol of Ce6 and 0.072 mmol of triethylamine (TEA) were mixed in 10 mL of dry dimethyl sulfoxide. Then, the mixture was stirred for 2 h at 37 °C. Later on, 0.10 mmol N-hydroxysuccinimide (NHS) and 0.12 mmol N,N′- dicyclohexylcarbodiimide (DCC) were added to the Ce6–TEA mixture. The solution was kept at 37 °C under gentle agitation in the dark for 12 h and filtered through a pore diameter of 0.22 μm. Finally, 1.0 g of APTMS-TiO_2_NRs was dispersed in 10 mL Na_2_CO_3_/NaHCO_3_ buffer (0.016 mol L^−1^, pH 9.0). Then, the solution with Ce6 obtained in the activation process was added and stirred in the dark for 2 h. The final products were dialyzed for 48 h against PBS 1X water to remove unreacted Ce6. The Ce6-conjugated material was centrifuged and dried at 40 °C for 72 h.

### 2.4. Materials’ Characterization

The N_2_ adsorption–desorption isotherms at 77 K were performed on a Micromeritics TriStar II 3020 apparatus. The specific surface areas were determined by the BET (Brunauer–Emmett–Teller) equation, using adsorption data over a relative pressure range between 0.05 and 0.3, and pore-size distributions were estimated using the BJH method. For surface area estimation and pore size distribution, samples were degassed at 120 °C for 3 h. The morphology and size of the nanostructure materials were examined by transmission electron microscopy (TEM) images, using a JEOL Model JEM-1200 EXII. XRD patterns were recorded on a Rigaku D/max-2500 diffractometer with the Cu Kα radiation at 40 kV and 100 mA. Infrared spectroscopy (FTIR) was carried out on a Perkin Elmer (Waltham, MA, USA) 1760-X spectrometer using a range of 4000–400 cm^−1^ and KBr pellets. The TGA experiments were conducted on a NETZSCH TG 209F3 using an O_2_ flow of 25 mL min^−1^ and a heating rate from 25 to 600 °C. Diffuse reflectance spectra recorded in the 200–900 nm spectral range were carried out to estimate the visible light response of Ce6-TiO_2_NRs in powder samples using a Thermo Scientific (Waltham, MA, USA) Evolution 260 UV–Vis spectrophotometer equipped with a Xenon flash lamp and a 60 mm integrating sphere with Spectralon^®^ coating.

### 2.5. Singlet Oxygen and Hydroxyl Radical Production

Singlet oxygen (^1^O_2_) and hydroxyl radical (HO•) generation was detected through commercially available fluorescent probes such as Singlet Oxygen Sensor Green (SOSG) and hydroxyphenyl fluorescein (HPF), respectively. In the first case, 5 µL of a 2 mmol L^−1^ methanol solution of SOSG was added to 100 µL of 2 mg mL^−1^ PBS suspension of pristine and Ce6-conjugated TiO_2_NRs in a 96-well plate. Control experiments were performed using phosphate-buffered saline (PBS) at pH 7.4 in the presence and absence of the SOSG methanolic solution to assess the intrinsic ability of these systems to induce ^1^O_2_ generation under the same experimental conditions. In addition, 100 µL nanomaterial dispersions were used without adding the SOSG reagent to confirm the absence of fluorescence emission signals from pristine and Ce6-conjugated TiO_2_NRs. Systems were irradiated with an LED light source (150 W m^−2^) in 3–60 min intervals, after which fluorescence intensity was read using excitation/emission filters of 488 nm and 525 nm, respectively. On the other hand, a similar protocol was used to detect HO•. Briefly, 5 µL of a 210 µmol L^−1^ aqueous solution of HPF was added to 100 µL of 2 mg mL^−1^ PBS suspension of pristine and Ce6-conjugated TiO_2_NRs in a 96-well plate. Control experiments were carried out using PBS at pH 7.4 in the presence and absence of the HPF aqueous solution to assess the intrinsic ability of these systems to induce HO• generation under the same experimental conditions. In addition, 100 µL nanomaterial dispersions were used without adding the HPF reagent to confirm the absence of fluorescence emission signals arising from NRs. Systems were irradiated with an LED light source (150 W m^−2^) in 3–60 min intervals, after which fluorescence intensity was read using excitation/emission filters of 488 nm and 525 nm, respectively. All experiments were carried out in triplicate using a POLARstar Omega multi-plate reader (BMG Labtech, Ortenberg, Germany).

### 2.6. Hemolysis

Fresh human blood samples were obtained from volunteers at the Department of Pharmacy, Faculty of Pharmacy, University of Concepción (Concepción, Chile) and collected in tubes with ethylenediamine tetraacetic acid (EDTA). In short, 4 mL of recently collected anticoagulated blood was centrifuged at 2500 rpm for 10 min and washed three times with PBS (1X, pH 7.4). Afterward, 140 μL of a 10% *v*/*v* base suspension of human red blood cells in PBS buffer were incubated on a rotary shaker for 30 min at 37 °C, after which solid suspensions (100, 300, 400, and 500 µg mL^−1^) were added. Pure 1X PBS buffer and deionized water were mixed with the human blood cell solution and used as negative and positive controls with 100% activity, respectively. The mixtures were incubated on a rotary shaker for 3 h at 37 °C before being centrifuged, and the absorbance of hemoglobin in the supernatants was measured using UV–Vis spectroscopy at 541 nm. Hemolysis percentages were calculated as follows:

(1) $$Hemolysys\left(\%\right)=\frac{Abs\;of\;sample-Abs\;of\;negative\;control}{Abs\;of\;positive\;control-Abs\;of\;negative}*100$$

### 2.7. Adsorption Efficiency and Loading Capacity

Drug adsorption was carried out by contacting 10 mg Ce6-TiO_2_NRs with 2 mL of DOX solution (0.2 mg mL^−1^ ethanolic solution) in an amber vial that was placed on a carousel rotating shaker for 24 h at a constant temperature (25 °C). Then, the sample was centrifuged, and the remaining concentration of the respective drug from the supernatant liquid was measured through a POLARstar Omega multi-plate reader (BMG Labtech) for DOX using previously recorded data from the calibration curves. The experiments were carried out in triplicate, and the drug loading capacity (LC) of Ce6-TiO_2_NRs was calculated according to Equation (2):

(2) $$LC\left(\frac{\mu g}{mg}\right)={(C}_{0}-C)\times 543.5\times V\times 10^{3}/M_{e}$$

where C_0_ = initial amount of DOX (M); C = amount of DOX in supernatant after encapsulation (M); 543.5 = molar mass of DOX; V = volume of DOX solution used (mL); and Me = amount of Ce6-TiO_2_NRs used for adsorption (mg).

### 2.8. In Vitro Drug Release Study

The DOX release from Ce6-TiO_2_NRs was measured using Spectra/Por^®^ dialysis membrane methods. To do this, the Ce6-TiO_2_NRs loaded with DOX (~11 mg) were dispersed in 22 mL of PBS (pH 7.4) and shaken at 37 °C under dark conditions for 24 h. At certain intervals, aliquots of the dialysate for DOX detection were taken and replaced with an equivalent volume of fresh PBS medium. The concentration of the released DOX was determined via a POLARstar Omega multi-plate reader (BMG Labtech) for doxorubicin based on the specific calibration curves.

### 2.9. Cell Viability Assays

The in vitro evaluation of the potential of the prepared nanomaterials for drug delivery and visible light-driven phototherapy applications was performed using a cellular model and CellTiter 96 ^®^ Aqueous One Solution Cell Proliferation Assay (MTS) reagent. For this purpose, approx. 5000 cells per well were cultured in 96-well plates using Dulbecco’s modified Eagle’s medium (DMEM) supplemented with 10% fetal bovine serum in an incubator containing 5% CO_2_ at 37 °C for 24 h. For phototoxicity evaluation, 100 µL of TiO_2_NR and Ce6-TiO_2_NR suspensions prepared in DMEM medium were added to the cells in the 20 to 300 µg mL^−1^ range. The suspensions were irradiated for 15 min with an LED light source (150 W m^−2^) and then incubated at 37 °C in 5% CO_2_ for up to 24 h. For cytotoxicity experiments, cells were contacted with 100 µL of the TiO_2_NR and Ce6-TiO_2_NR suspensions prepared in DMEM medium at the same concentration ranges and maintained in dark conditions at 37 °C in 5% CO_2_ for 24 h. For chemo-cytotoxicity experiments, cells were contacted with 100 µL of preloaded TiO_2_NR and Ce6-TiO_2_NR solid suspensions with DOX, which were prepared in a DMEM medium at a range of 100 to 300 µg mL^−1^ and incubated at 37 °C in 5% CO_2_ for 12 and 24 h. For combined therapy experiments, cells were contacted with 100 µL of preloaded black suspensions with DOX, which were prepared in DMEM medium in the range of 100 to 300 µg mL^−1^ and then incubated in the dark at 37 °C for 5% CO_2_ for 12 h. Afterwards, the medium was irradiated for 15 min with LED light (150 W m^−2^) and re-incubated at 37 °C for 12 h, completing a total of 24 h of incubation. In every case, control experiments were conducted without the suspensions, where the cells were contacted with 100 µL of 0.1% Triton solution as death C (+) and 100 µL of fresh DMEM culture medium as survival C (−). After each treatment, cell viability was assessed using the MTS reagent according to the manufacturer’s instructions.

### 2.10. Immunofluorescence Staining

A total of 180,000 HeLa cells were seeded onto slides in a 6-well plate using DMEM supplemented with 10% fetal bovine serum. The plate was then incubated in an atmosphere containing 5% CO_2_ at 37 °C for 24 h. Afterward, the cells were contacted with 2 mL of Ce6-TiO_2_NRs, which were prepared in DMEM medium at a suspension of 100 µg mL^−1^. Control samples were also included in the absence of the solids. Suspensions of Ce6-TiO_2_NRs were irradiated for 15 min with an LED light source (150 W m^−2^), and in parallel, a control group was kept in dark conditions. Afterward, cells were incubated for 8 h, fixed by contact with a 3.7% solution of paraformaldehyde in DMEM for 20 min, and then permeabilization was carried out using PBS 1X with 0.1% Triton-X100. Subsequently, non-specific binding sites were blocked with a 0.5% BSA solution in PBS, and immunofluorescence staining was performed. For this purpose, the cells were incubated overnight at 4 °C with primary antibodies, such as anti-DNA-RNA damage (ab62623), anti-4-hydroxynonenal (ab46545), and anti-cleaved PARP1 (9541S) antibodies, all diluted 1:50. Cells were extensively washed with PBS and later incubated with anti-Mouse Alexa Fluor^®^ 488 and anti-Rabbit Alexa Fluor^®^ 555 secondary antibodies diluted 1:700 for 2 h in the dark at room temperature. Once the incubation was completed, the slides were rewashed with PBS and mounted on slides using VECTASHIELD^®^ Antifade Mounting Medium with DAPI. Finally, cells were observed for photographic recording with an EVOS fluorescence microscope using a 40× objective.

## 3. Results and Discussion

### 3.1. Characterization of Nanomaterials

**Figure 1:**
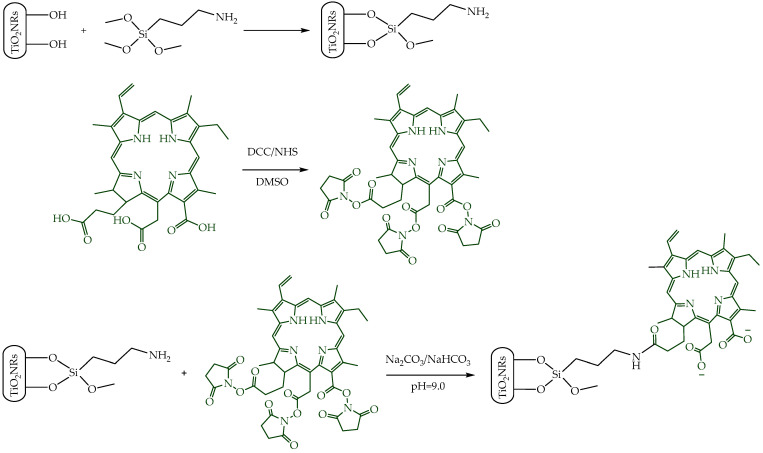
Illustration of the synthetic procedure of Ce6-conjugated titania nanorods.

**Figure 2:**
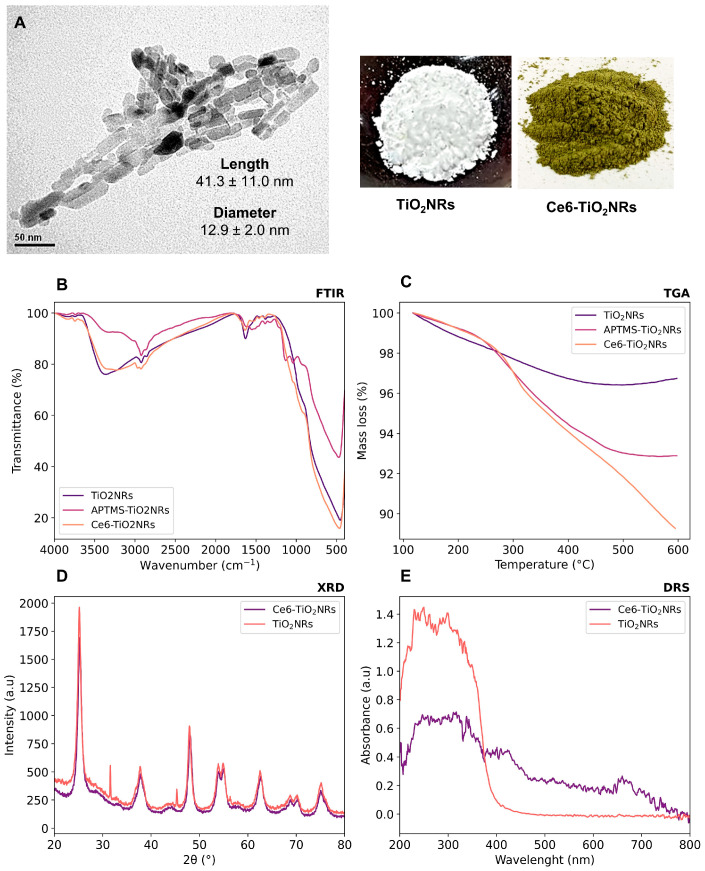
Characterization of the synthesized nanomaterials by (**A**) TEM, (**B**) FTIR, (**C**) TGA, (**D**) XRD, and (**E**) DRS.

Pristine TiO_2_NRs were synthesized using hydrogen titanate nanotubes as a precursor material; these were calcined at 500 °C. Subsequently, a silane-mediated conjugation strategy was carried out to obtain Ce6-TiO_2_NRs (Figure 1). According to previous reported results by Gatusso et al., conjugation is expected to occur through the most available carboxyl group, which is the one that connects to the Ce6 core with an ethylene spacer. This carboxyl group is oriented outside the plane of the Ce6 core, whereas the other two carboxyls lie in the molecular plane in positions that are less accessible for conjugation reactions [19]. The pristine and modified TiO_2_ solids were characterized through TEM, FTIR, TGA, XRD, and DRS analyses. TEM micrographs confirmed the formation of TiO_2_NRs (Figure 2A), with a length and diameter of 41.3 ± 11.0 y 12.9 ± 2.0 nm, respectively. The covalent immobilization of Ce6 on the TiO_2_NRs’ surface was verified by FTIR spectra (Figure 2B). The pristine TiO_2_NR spectrum shows characteristic bands at 3394 cm^−1^ related to symmetric and asymmetric stretching vibrations of surface Ti-OH and adsorbed H_2_O molecules, 1634 cm^−1^ associated with –OH bending vibrations, and from 1000 to 400 cm^−1^, corresponding to the Ti-O stretching and Ti-O-Ti bridging stretching modes [20]. The FTIR spectrum for the APTMS-modified TiO_2_NRs shows a characteristic signal at 2925 cm^−1^ corresponding to C-H stretching vibrations, the Si-O stretching modes around 1050 cm^−1^, and finally, the signal attributed to N-H bending of the APTMS terminal group at 1560 cm^−1^ [21]. Regarding the Ce6-conjugated TiO_2_NRs, a new, weak band is observed at 1523 cm^−1^, corresponding to the C=N stretching vibration of pyrrole, whereas the C=O band for Ce6 appears at 1637 cm^−1^. This band is shifted from the C=O signal of free Ce6 (1686 cm^−1^), which confirms the covalent coupling to TiO_2_NRs [11]. Finally, the band that indicates the presence of the C-N bond was detected at 1384 cm^−1^. The APTMS and Ce6 conjugation degrees were estimated from thermogravimetric analysis (TGA) experiments (Figure 2C). In this regard, the difference in the mass loss between pristine and modified TiO_2_NRs resulted in conjugation degrees of 3.9% for APTMS and 3.6% for Ce6. XRD patterns were similar for pristine and modified TiO_2_ materials (Figure 2D). The observed diffraction peaks appeared at 25.28° (1 0 1), 37.80° (0 0 4), 48.00° (2 0 0), 53.85° (1 0 5), 55.08° (2 1 1), 62.70° (2 0 4), and 75.07° (2 1 5), corresponding to the anatase phase of TiO_2_ (JCPDS-ICDD card: 21-1272) [22]. UV–Vis diffuse reflectance spectra for pristine TiO_2_NRs and Ce6-TiO_2_NRs show the typical absorption band of anatase TiO_2_ at wavelengths <400 nm. Meanwhile, the Ce6-conjugated TiO_2_NRs show new bands in the visible region where Ce6 absorbs. These results support the binding of the photosensitizer and show the change in the photo response towards the visible light spectrum of the Ce6-conjugated materials.

### 3.2. Generation of ROS

**Figure 3:**
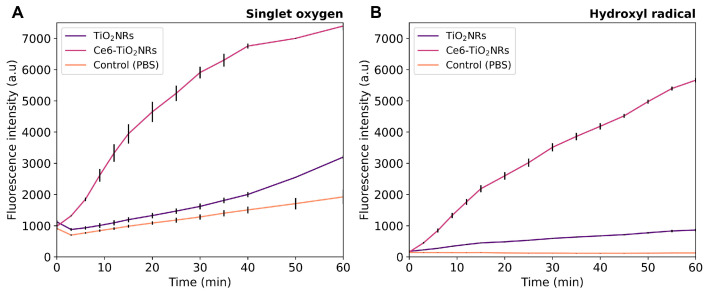
(**A**) HO• and (**B**) ^1^O_2_ generation experiments in pristine TiO_2_NRs and Ce6-TiO_2_NRs under light LED irradiation (60 min, 150 W m^−2^). Experiments were carried out in triplicate using 2 mg mL^−1^ suspensions using the HPF (10 µM) and SOSG (40 µM) fluorescent probes, respectively. Fluorescence was measured using excitation/emission wavelengths of 488/525 nm.

The possible visible light-driven PDT ability of the pristine TiO_2_NRs and Ce6-TiO_2_NRs was evaluated by measuring the generation of ^1^O_2_ and HO• in PBS dispersions of these nanomaterials under visible light irradiation, using commercial fluorescent probes for each ROS detection. In these experiments, the intensity of the fluorescence emission is proportional to the quantity of ROS generated. Control experiments were conducted using only PBS and the ROS probes under identical conditions. Parallel experiments without the sensor were also conducted to confirm the absence of intrinsic fluorescence emission in the material dispersions (data provided as Supporting Information). Ce6-TiO_2_NRs showed a higher capacity to produce HO• and ^1^O_2_ than the pristine materials or the control experiments, thus supporting the photodynamic properties of the conjugate system (Figure 3). The simultaneous generation of HO• and ^1^O_2_ indicates that type I and II photochemical reactions are induced by the Ce6-TiO_2_NR system [23]. These results are auspicious, as they confirm that the photodynamic properties of Ce6 were not compromised by conjugation onto the surface of TiO_2_NRs, which is valuable for using the conjugated material in visible light-induced PDT applications.

### 3.3. Hemolytic Activity

**Figure 4:**
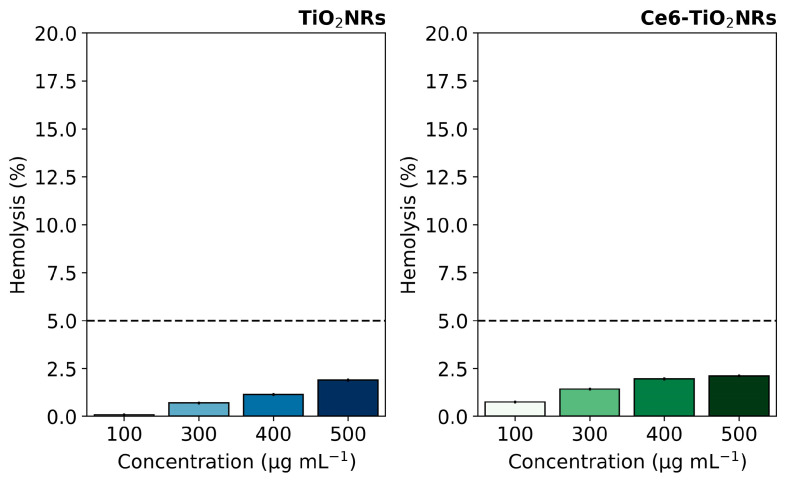
Hemolysis percentages induced by pristine TiO_2_NRs and Ce6-TiO_2_NRs. Dashed line correspond to the maximum value for non-hemolytic activity.

Hemolytic activity assays were conducted to evaluate the intrinsic safety of the studied nanomaterials in their interaction with blood components. This evaluation is crucial for further developing nanoparticle-based therapeutic applications, where materials are expected to be compatible with living blood tissue and can be delivered to the site of action without undesirable effects. The hemolytic activity of pristine TiO_2_NR and Ce6-TiO_2_NR materials was evaluated by contacting suspensions of the solid materials with red blood cells (RBC). The test measured the free hemoglobin due to erythrocyte lysis after contact with pristine TiO_2_NRs and Ce6-TiO_2_NRs at the concentrations used in cell viability experiments. The negative control (C−) was carried out with PBS 1X in contact with RBC, and the positive control (C+) (100% hemolytic activity) was evaluated using water. TiO_2_NRs and Ce6-TiO_2_NRs showed a hemolysis percentage of less than 5% in the entire concentration range assayed, which indicates the absence of harmful hemolytic activity (Figure 4). These findings are within the ranges established by the ISO 10993-4 standard [24], which specifies that any system with a hemolysis percentage lower than 5% is suitable for pursuing applications associated with drug delivery. Therefore, pristine TiO_2_NRs and Ce-TiO_2_NRs are safe for exploring future biomedical applications.

### 3.4. Cytotoxicity and Phototoxicity Assays

**Figure 5:**
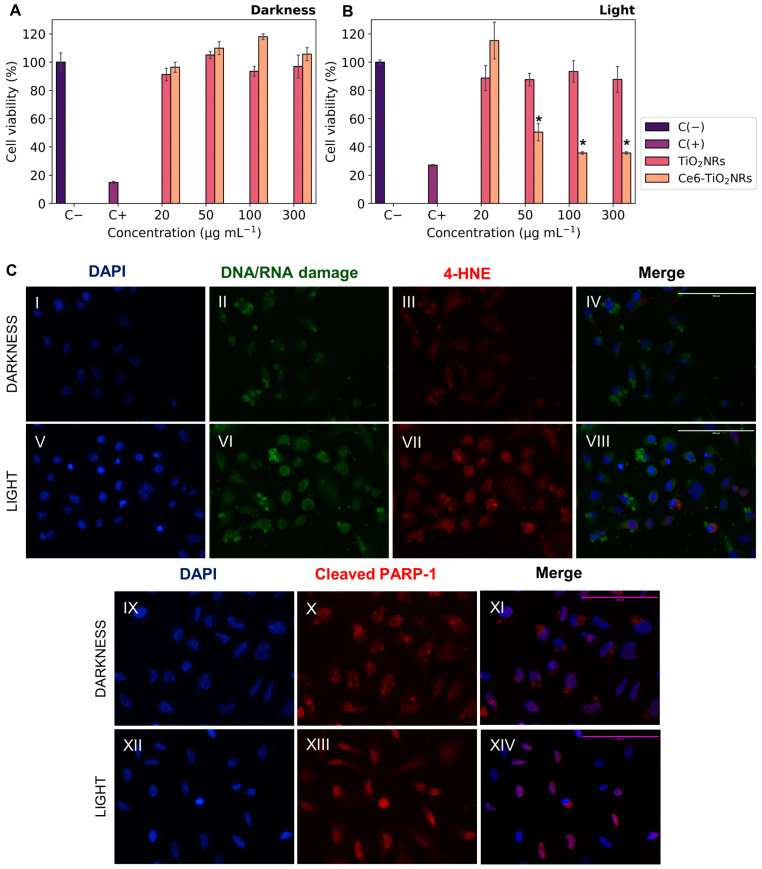
Cell viability experiments with HeLa cells contacted with Ce6-TiO_2_NRs: (**A**) cytotoxicity (darkness); (**B**) irradiation with LED light (15 min, 150 W m^−2^) (phototoxicity). The results were expressed as percentages referred to untreated cells and presented as mean ± SD, n = 3. Statistically significant differences with the control (−) are marked with * and correspond to *p* < 0.050. (**C**) Immunofluorescence assays with HeLa cells in the presence of Ce6-TiO_2_NRs (100 µg mL^−1^) under darkness and irradiation with LED light (15 min, 150 W m^−2^). DAPI nuclear detection was used (**C(II)**,**C(VI)**) with a green channel for DNA/RNA damage and (**C(III)**,**C(VII)**) a red channel for membrane damage (**C(X)**,**C(XIII)**), with a red channel for apoptosis. Images were collected with the 40× objective.

The cytotoxicity of pristine TiO_2_NRs and Ce6-TiO_2_NRs in dark conditions was evaluated by incubating HeLa cells with suspensions of these materials at increasing concentrations. Cell viability was measured using the MTS metabolic activity assay (Figure 5A). Both pristine TiO_2_NRs and Ce6-TiO_2_NRs resulted in negligible effects on cell viability after 24 h of incubation in the dark, in the entire range of assayed dispersions. These findings confirm the safety of both materials in dark conditions. As control experiments, the phototoxicity of LED light was evaluated by incubating HeLa cells and irradiating the samples with visible LED light (15 min, 150 W m^−2^) in the absence of nanomaterials (see Supporting Information). The LED light did not display toxicity in the HeLa cells. The phototoxicity of pristine TiO_2_NRs and Ce6-TiO_2_NRs was evaluated by incubating HeLa cells with suspensions of increasing dispersion of these nanomaterials and irradiating the samples with visible LED light (15 min, 150 W m^−2^). Cell viability was measured after 24 h of subsequent incubation using the MTS assay (Figure 5B). Irradiated systems containing TiO_2_NRs showed negligible changes in cell viability compared with the survival control, which agrees with the absence of photodynamic activity in this system (Figure 3) [25]. On the other hand, the irradiated systems containing Ce6-TiO_2_NRs showed a major decrease in cell viability in the whole range of assayed dispersions. The maximum effect was achieved by 100 µg mL^−1^ suspensions, with a cell viability of 35%. According to ISO10993-5 classification, these findings indicate that Ce6-TiO_2_NRs are potent phototoxic compounds (<40% viability), which is a highly auspicious outcome for the purpose of the present study [26].

We performed indirect immunofluorescence tests to evaluate the effects of visible light-mediated ROS release on HeLa cells incubated with Ce6-TiO_2_NRs dispersions at 100 µg mL^−1^. We evaluated 8-hydroxy-2′-deoxyguanosine (8-OHdG), a biomarker of oxidative DNA damage, and the effect of ROS on cell membranes by identifying 4-hydroxynonenal (4-HNE), one of the leading secondary products generated in lipid peroxidation, considered a marker and initiator of oxidative damage. Furthermore, it was interesting to evaluate the induction of a cell death mechanism such as apoptosis, for which the cleaved PARP-1 antibody was used as the primary antibody. Control experiments were carried out with the cells without the Ce6-TiO_2_NR suspensions to evaluate the basal response of the antibodies in dark and irradiation conditions. Additional controls without solids or primary and secondary antibodies were included in the Supporting Information to assess the initial response corresponding to the antibody dilution buffer. The findings from the Ce6-TiO_2_NR 100 µg mL^−1^ suspensions (Figure 5C) demonstrate higher fluorescence intensities in the green 8-OHdG and red channels for 4-HNE after photoactivation. This suggests that systems exposed to LED light (Figure 5C(VI,VII)) increase the levels of the assessed markers compared to the response in the dark (Figure 5C(II,III)) and control experiments, which account for the cell damage induced by ROS after irradiation with visible LED light, affecting nucleic acids and cell membranes according to the green and red channel results, respectively.

Additionally, Figure 5C(XIII) reveals an increase in fluorescence intensity within the nuclear region compared to dark controls (Figure 5C(X)), implying the existence of this cellular death pathway. This is in line with the decrease in cell viability evidenced in the phototoxicity results (Figure 5B). Our findings demonstrate the potential of Ce6-TiO_2_NRs for PDT under visible light irradiation. Nonetheless, this activity might benefit from combination with another strategy, such as chemotherapy [27]. Therefore, in the following sections, we present the evaluation of Ce6-TiO_2_NRs as nanoplatforms for combined chemo-PDT applications.

### 3.5. Drug Loading Capacity and DOX In Vitro Release

The drug loading capacity of Ce6-TiO_2_NRs was evaluated using DOX as a chemotherapeutic drug model. DOX, a member of the anthracycline family, is a prominent chemotherapeutic agent utilized extensively for treating various cancers. Numerous studies have demonstrated its efficacy in exploring combined cancer therapy approaches [28,29]. An ethanolic DOX solution was contacted with Ce6-TiO_2_NRs under constant stirring for 24 h. The loading capacity of the Ce6-TiO_2_NRs was determined from the difference between the initial and final concentrations in the supernatant solution after the contact time, resulting in a total adsorption capacity of 16 μg_DOX_/mg_Ce6-TiO2NRs_.

**Figure 6:**
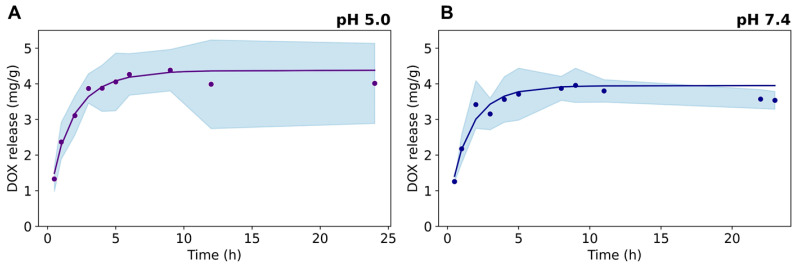
Release kinetics of DOX from Ce6-TiO_2_NRs at pH (**A**) 5.0 and (**B**) 7.4.

DOX release was evaluated from dialysis experiments at pH 7.4 and pH 5.0, which mimic the acidity conditions of external and internal tumoral cell microenvironments, respectively (Figure 6). The drug release kinetics show a fast DOX release in the first 5 h, followed by a steady behavior up to 24 h at both pH conditions. A slightly higher first-order kinetic constant was found for DOX release at pH 5.0 (data provided as Supporting Information), arising from the electrostatic repulsion between the positively charged drug and the cationic ≡Ti–OH_2_^+^ surface groups present in the TiO_2_NRs (isoelectric point at pH = 5.8 [30]), which promotes the fast release of the sorbed drug on the nanocarrier. At pH 7.4, the Ce6-TiO_2_NRs’ surface is expected to be negatively charged, thus strengthening the electrostatic interactions governing the adsorption of the drug onto the nanomaterial.

### 3.6. Photo-Chemo Toxicity Experiments

**Figure 7:**
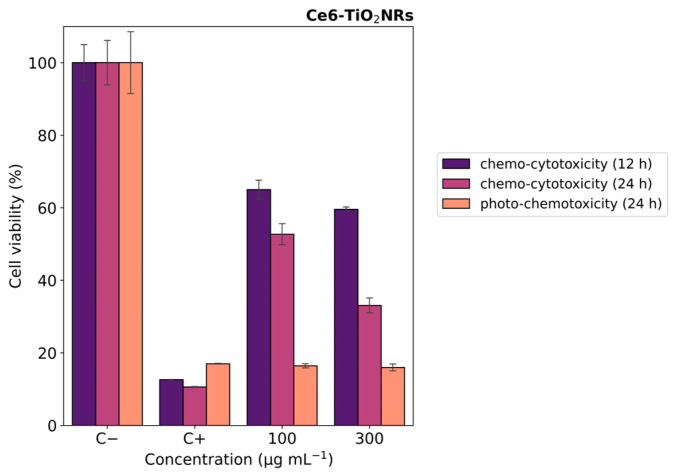
Cell viability experiments with HeLa cells contacted with DOX:Ce6-TiO_2_NR formulations. Blue color indicates 12 h, purple color indicates 24 h (darkness) (chemo-cytotoxicity), and orange color indicates 12 h darkness, irradiation with LED light (15 min, 150 W m^−2^), and darkness up to 24 h (photo-chemotoxicity). The results were expressed as percentages referred to untreated cells and presented as mean ± SD, n = 3.

In vitro photo-chemotoxicity studies were carried out with HeLa cells to test the capacity of the Ce6-TiO_2_NRs conjugate material to serve as a dual platform for chemo-PDT applications. For this, Hela cells were incubated with a formulation of DOX:Ce6-TiO_2_NRs (100 and 300 µg mL^−1^) corresponding to nominal DOX concentrations from 1.60 to 4.8 µg mL^−1^. Figure 7 shows the MTS results after 12 h of contact with suspensions in dark conditions. After 12 h of incubation, the cells treated with DOX-loaded materials exhibited moderate decreases in cell viability at both dispersion concentrations. These findings align with the expected cytotoxic effects of DOX release from the nanomaterial. After 24 of incubation, the systems treated with the formulation of DOX:Ce6-TiO_2_NRs exerted a progressive decrease in cell viability in a concentration-dependent manner (Figure 7 purple color). Combined photo-chemotoxicity experiments were carried out by incubating HeLa cells with DOX-loaded dispersions of the DOX:Ce6-TiO_2_NRs for 12 h, followed by irradiation with visible LED light (15 min, 150 W m^−2^) and 12 h of further incubation (24 h in total) (Figure 7 orange color). These findings are consistent with earlier studies reporting the development of a multimodal nanocarrier based on mesoporous silica nanoparticles (MSNs) for the co-delivery of photosensitizer Ce6 and DOX as a chemotherapeutic agent for combined cancer therapy [31].

## 4. Conclusions

Chlorin e6-conjugated mesoporous TiO_2_ nanorods were prepared, characterized, and evaluated as novel nanoplatforms for visible light-mediated dual photo-chemotherapy. Our results demonstrated that the covalent conjugation of Chlorin e6 to mesoporous TiO_2_ nanorods conferred photodynamic properties to the materials under visible light irradiation while retaining suitable capacity for drug loading and release. Chlorin e6-mesoporous TiO_2_ nanorods loaded with a model anticancer drug exhibited a synergic chemo- and phototoxic effect against HeLa cells. Our findings indicate that combining doxorubicin with phototherapy by irradiation with Chlorin e6-mesoporous TiO_2_ nanorods could improve the efficacy against the viability of HeLa cells and point to possible alternatives to overcome the limitations associated with monotherapy.

## Data Availability

Data are contained within the article.

## Supplementary Materials

The following supporting information can be downloaded at: https://www.mdpi.com/article/10.3390/nano14110933/s1, Figure S1. Spectra of the white LED used in the PDT and PCT experiments. Figure S2. FTIR peak assignment for pristine and Ce6-conjugated TiO_2_NRs. Figure S3. Control measurements of hydroxyl radical and singlet oxygen generation experiments with TiO_2_NRs and Ce6-TiO_2_NRs under light LED irradiation (15 min, 150 W m^−2^). Experiments were carried out with 2 mg mL^−1^ suspensions using the HPF (10 μM) and SOSG (40 μM) fluorescent probes. Fluorescence was measured using excitation/emission wavelengths of 488/525 nm. Figure S4. Indirect immunofluorescence control experiments in darkness and light. Intrinsic fluorescence of the antibody dilution buffer in their presence and absence. DAPI nuclear detection was used—Images were collected with the 40× objective. Figure S5. Cell viability experiments with HeLa cells under irradiation with LED light (15 min, 150 W m^−2^) (phototoxicity). The results were expressed as percentages referred to untreated cells and presented as mean ± SD, n = 3. Statistically significant differences with the control (-) are marked with * and correspond to *p* < 0.050. Table S1. First-order kinetic model equation for the release of DOX from Ce6-TiO_2_NRs.

## References

[1] Sung H., Ferlay J., Siegel R.L., Laversanne M., Soerjomataram I., Jemal A., Bray F., Bsc M.F.B., Me J.F., Soerjomataram M.I. (2021). Global Cancer Statistics 2020: GLOBOCAN Estimates of Incidence and Mortality Worldwide for 36 Cancers in 185 Countries. CA A Cancer J. Clin..

[2] Cheng L., Wang C., Feng L., Yang K., Liu Z. (2014). Functional Nanomaterials for Phototherapies of Cancer. Chem. Rev..

[3] Chen J., Fan T., Xie Z., Zeng Q., Xue P., Zheng T., Chen Y., Luo X., Zhang H. (2020). Advances in nanomaterials for photodynamic therapy applications: Status and challenges. Biomaterials.

[4] Liu M., Li C. (2020). Recent Advances in Activatable Organic Photosensitizers for Specific Photodynamic Therapy. ChemPlusChem.

[5] Park J., Lee Y.-K., Park I.-K., Hwang S.R. (2021). Current Limitations and Recent Progress in Nanomedicine for Clinically Available Photodynamic Therapy. Biomedicines.

[6] Pourmadadi M., Rajabzadeh-Khosroshahi M., Eshaghi M.M., Rahmani E., Motasadizadeh H., Arshad R., Rahdar A., Pandey S. (2023). TiO_2_-based nanocomposites for cancer diagnosis and therapy: A comprehensive review. J. Drug Deliv. Sci. Technol..

[7] Sargazi S., Simge E.R., Gelen S.S., Rahdar A., Bilal M., Arshad R., Pandey S. (2022). Application of titanium dioxide nanoparticles in photothermal and photodynamic therapy of cancer: An updated and comprehensive review. J. Drug Deliv. Sci. Technol..

[8] Ziental D., Czarczynska-Goslinska B., Mlynarczyk D.T., Glowacka-Sobotta A., Stanisz B., Goslinski T., Sobotta L. (2020). Titanium Dioxide Nanoparticles: Prospects and Applications in Medicine. Nanomaterials.

[9] Hak A., Ali M.S., Sankaranarayanan S.A., Shinde V.R., Rengan A.K. (2023). Chlorin e6: A Promising Photosensitizer in Photo-Based Cancer Nanomedicine. ACS Appl. Bio Mater..

[10] Zhao X., Shen R., Bao L., Wang C., Yuan H. (2020). Chitosan derived glycolipid nanoparticles for magnetic resonance imaging guided photodynamic therapy of cancer. Carbohydr. Polym..

[11] Youssef Z., Jouan-Hureaux V., Colombeau L., Arnoux P., Moussaron A., Baros F., Toufaily J., Hamieh T., Roques-Carmes T., Frochot C. (2018). Titania and silica nanoparticles coupled to Chlorin e6 for anti-cancer photodynamic therapy. Photodiagnosis Photodyn. Ther..

[12] Jiao X., Zhang W., Zhang L., Cao Y., Xu Z., Kang Y., Xue P. (2020). Rational design of oxygen deficient TiO_2−x_ nanoparticles conjugated with chlorin e6 (Ce6) for photoacoustic imaging-guided photothermal/photodynamic dual therapy of cancer. Nanoscale.

[13] Rejinold N.S., Choi G., Choy J.-H. (2020). Recent trends in nano photo-chemo therapy approaches and future scopes. Coord. Chem. Rev..

[14] Nešić M., Žakula J., Korićanac L., Stepić M., Radoičić M., Popović I., Šaponjić Z., Petković M. (2017). Light controlled metallo-drug delivery system based on the TiO_2_-nanoparticles and Ru-complex. J. Photochem. Photobiol. A Chem..

[15] Salahuddin N., Abdelwahab M., Gaber M., Elneanaey S. (2020). Synthesis and Design of Norfloxacin drug delivery system based on PLA/TiO_2_ nanocomposites: Antibacterial and antitumor activities. Mater. Sci. Eng. C.

[16] Kafshgari M.H., Mazare A., Distaso M., Goldmann W.H., Peukert W., Fabry B., Schmuki P. (2019). Intracellular Drug Delivery with Anodic Titanium Dioxide Nanotubes and Nanocylinders. ACS Appl. Mater. Interfaces.

[17] Torres C.C., Campos C.H., Diáz C., Jiménez V.A., Vidal F., Guzmán L., Alderete J.B. (2016). PAMAM-grafted TiO_2_ nanotubes as novel versatile materials for drug delivery applications. Mater. Sci. Eng. C.

[18] Jiménez V.A., Moreno N., Guzmán L., Torres C.C., Campos C.H., Alderete J.B. (2020). Visible-light-responsive folate-conjugated titania and alumina nanotubes for photodynamic therapy applications. J. Mater. Sci..

[19] Gattuso H., Monari A., Marazzi M. (2017). Photophysics of chlorin e6: From one- and two-photon absorption to fluorescence and phosphorescence. RSC Adv..

[20] Praveen P., Viruthagiri G., Mugundan S., Shanmugam N. (2014). Structural, optical and morphological analyses of pristine titanium di-oxide nanoparticles–Synthesized via sol–gel route. Spectrochim. Acta Part A Mol. Biomol. Spectrosc..

[21] Suddai A., Nuengmatcha P., Sricharoen P., Limchoowong N., Chanthai S. (2018). Feasibility of hard acid–base affinity for the pronounced adsorption capacity of manganese(ii) using amino-functionalized graphene oxide. RSC Adv..

[22] Qaid S.M.H., Ghaithan H.M., Bawazir H.S., Bin Ajaj A.F., AlHarbi K.K., Aldwayyan A.S. (2023). Successful Growth of TiO_2_ Nanocrystals with {001} Facets for Solar Cells. Nanomaterials.

[23] Baptista M.S., Cadet J., Di Mascio P., Ghogare A.A., Greer A., Hamblin M.R., Lorente C., Nunez S.C., Ribeiro M.S., Thomas A.H. (2017). Type I and Type II Photosensitized Oxidation Reactions: Guidelines and Mechanistic Pathways. Photochem. Photobiol..

[24] (2009). Biological Evaluation of Medical Devices: Selection of Tests for Interactions with Blood.

[25] Jukapli N.M., Bagheri S. (2016). Recent developments on titania nanoparticle as photocatalytic cancer cells treatment. J. Photochem. Photobiol. B Biol..

[26] (2009). Biological Evaluation of Medical Devices: Tests for In Vitro Cytotoxicity.

[27] Denkova A.G., de Kruijff R.M., Serra-Crespo P. (2018). Nanocarrier-Mediated Photochemotherapy and Photoradiotherapy. Adv. Healthc. Mater..

[28] Cacaccio J.C., Durrani F.A., Missert J.R., Pandey R.K. (2022). Photodynamic Therapy in Combination with Doxorubicin Is Superior to Monotherapy for the Treatment of Lung Cancer. Biomedicines.

[29] Sadeghloo A.Y., Khorsandi K., Kianmehr Z. (2020). Synergistic effect of photodynamic treatment and doxorubicin on triple negative breast cancer cells. Photochem. Photobiol. Sci..

[30] McNamee C.E., Tsujii Y., Matsumoto M. (2005). Physicochemical Characterization of an Anatase TiO_2_ Surface and the Adsorption of a Nonionic Surfactant:  An Atomic Force Microscopy Study. Langmuir.

[31] Sun J.-H., Zhang W., Zhang D.-Y., Shen J., Tan C.-P., Ji L.-N., Mao Z.-W. (2018). Multifunctional mesoporous silica nanoparticles as efficient transporters of doxorubicin and chlorin e6 for chemo-photodynamic combinatorial cancer therapy. J. Biomater. Appl..

