# Supplementary material for: Chlorin e6-Conjugated Mesoporous Titania Nanorods as Potential Nanoplatform for Photo-Chemotherapy
Source: Nanomaterials (Basel). 2024 May 25;14(11):933. doi: 10.3390/nano14110933 (PMC11173822; doi:10.3390/nano14110933)
Supplement: Supplementary file 1 [file nanomaterials-14-00933-s001.zip › nanomaterials-2992388-supplementary.pdf]

nanomaterials

Article

Chlorin e6-Conjugated Mesoporous Titania Nanorods as Potential Nanoplatform for Photo-Chemotherapy

Estefanía Vélez-Peña [1], Verónica A. Jiménez [2], Joaquín Manzo-Merino [3], Joel B. Alderete [4,*] and Cristian H. Campos [1,*]

1 Departamento de Fisicoquímica, Facultad de Ciencias Químicas, Universidad de Concepción, Edmundo Larenas 129, Casilla 160-C, Concepción 4070371, Chile; evelez@udec.cl
2 Departamento de Ciencias Químicas, Facultad de Ciencias Exactas, Universidad Andres Bello, Sede Concepción, Autopista Concepción-Talcahuano 7100, Talcahuano 4300866, Chile; veronica.jimenez@unab.cl
3 Facultad de Ciencias Químicas, Benemérita Universidad Autónoma de Puebla, Puebla 72570, Mexico; jmanzomerino@gmail.com
4 Instituto de Química de Recursos Naturales (IQRN), Universidad de Talca, Avenida Lircay S/N, Casilla 747, Talca 3341717, Chile
* Correspondence: (JBA) joel.alderete@utalca.cl and (CHC) ccampos@udec.cl

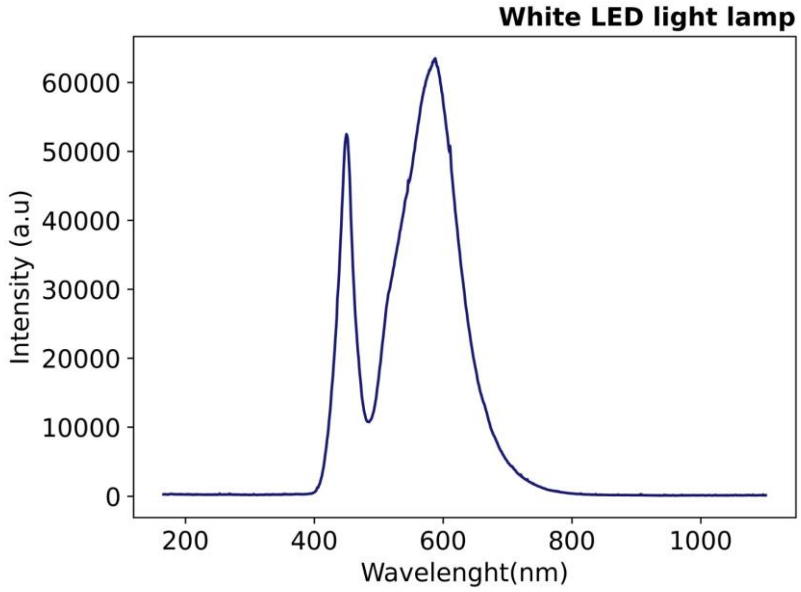

Figure S1. Spectra of the white LED used in the PDT and PCT experiments.

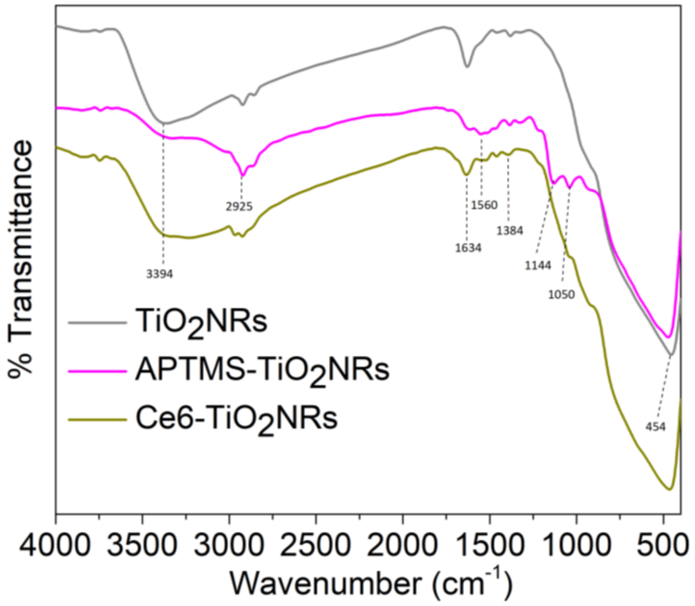

Figure S2. FTIR peak assignment for pristine and Ce6-conjugated TiO_2NRs.

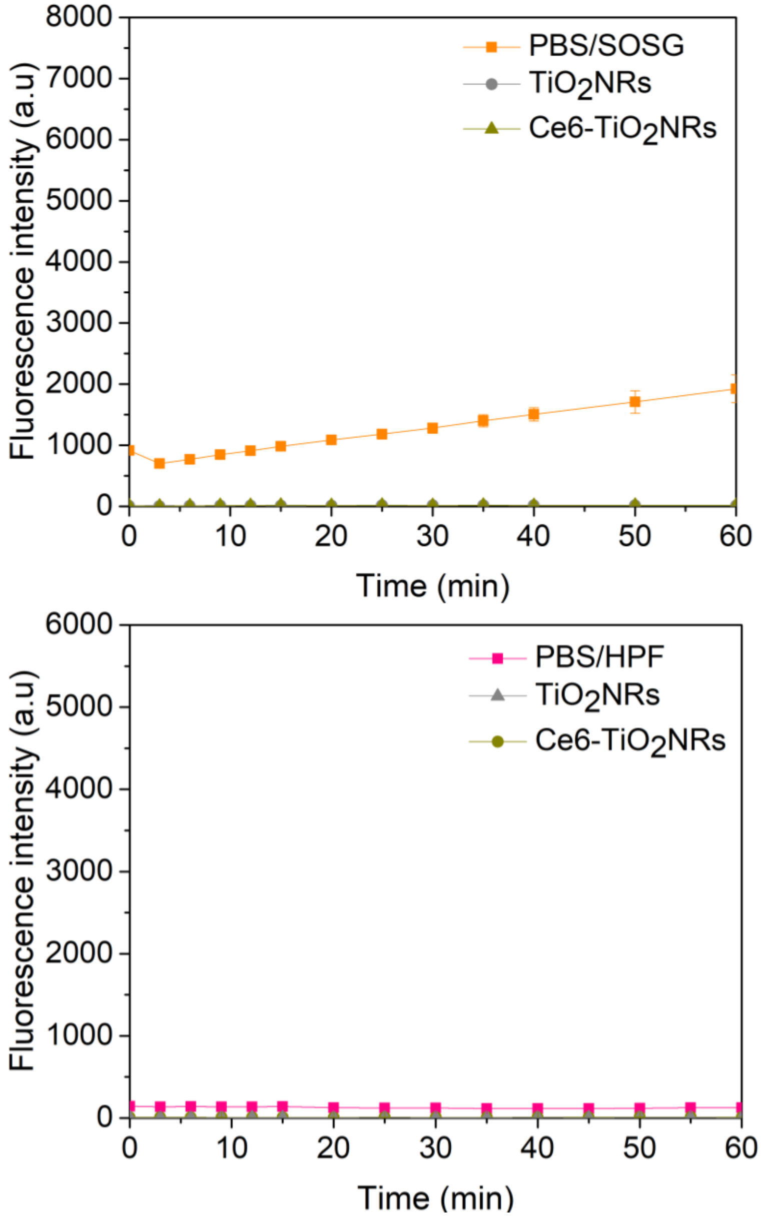

Figure S3. Control measurements of hydroxyl radical and singlet oxygen generation experiments with TiO_2NRs and Ce6-TiO_2NRs under light LED irradiation (15 min, 150 W m^{-2}). Experiments were carried out with 2 mg mL^{-1} suspensions using the HPF (10 μM) and SOSG (40 μM) fluorescent probes. Fluorescence was measured using excitation/emission wavelengths of 488/525 nm.

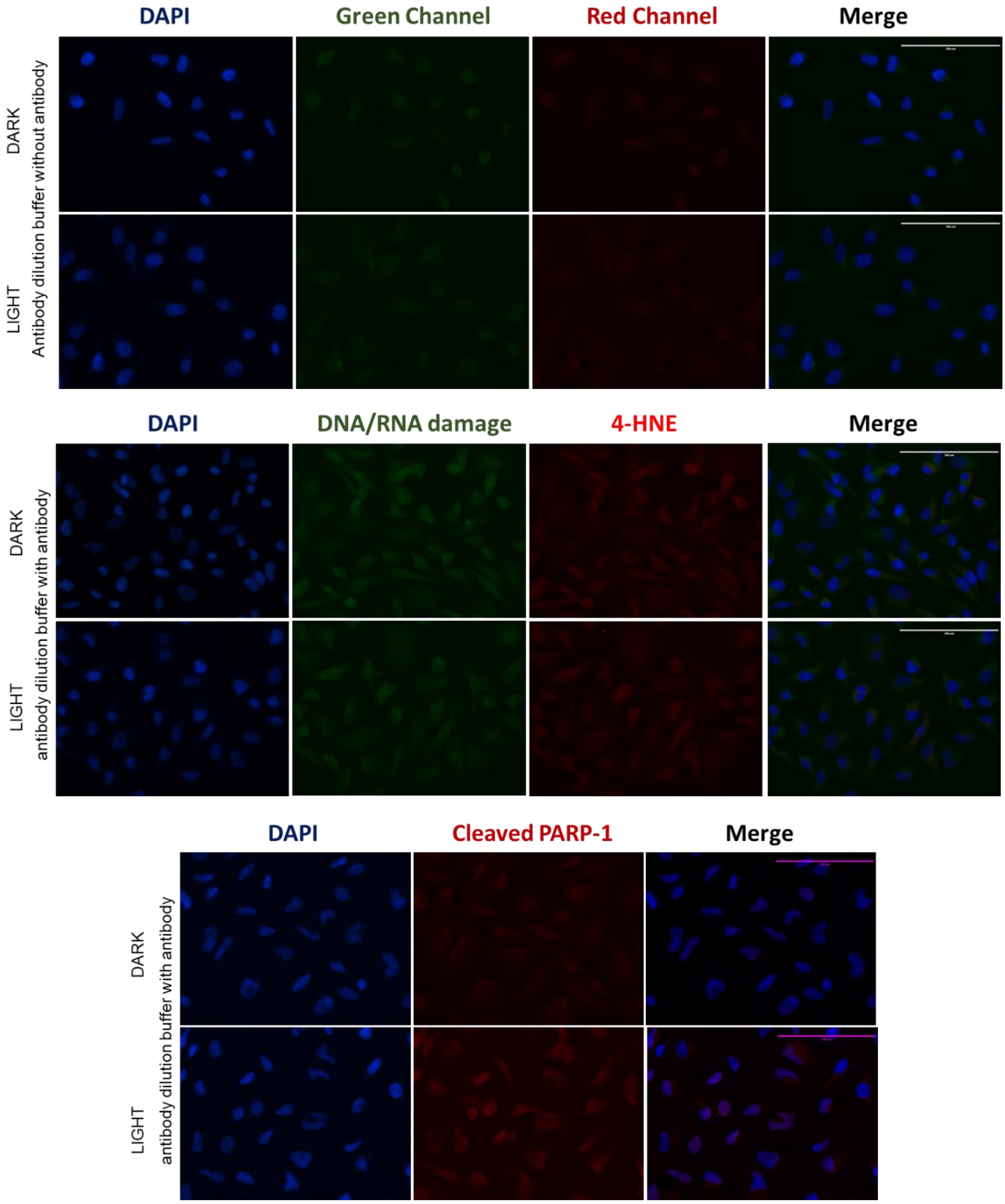

Figure S4. Indirect immunofluorescence control experiments in darkness and light. Intrinsic fluorescence of the antibody dilution buffer in their presence and absence. DAPI nuclear detection was used—Images were collected with the 40X objective.

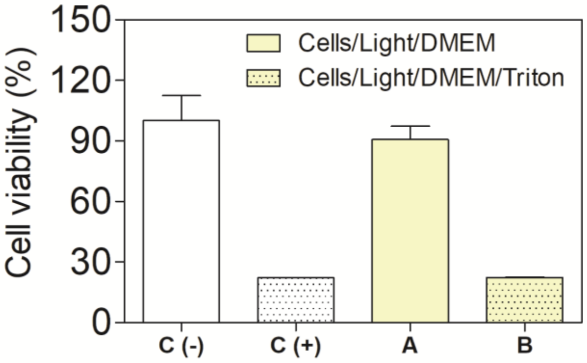

Figure S5. Cell viability experiments with HeLa cells under irradiation with LED light (15 min, 150 W m^{-2}) (phototoxicity). The results were expressed as percentages referred to untreated cells and presented as mean ± SD, n=3. Statistically significant differences with the control (-) are marked with * and correspond to $p < 0.050$.

Table S1. First-order kinetic model equation for the release of DOX from Ce6-TiO_2NRs

pH	k (min^{-1})	R^2	DOX released (mg L^{-1})
5.0	0.175	0.9782	4.79
7.4	0.096	0.9825	3.61
